# Properties and Differential Expression of H^+^ Receptors in Dorsal Root Ganglia: Is a Labeled-Line Coding for Acid Nociception Possible?

**DOI:** 10.3389/fphys.2021.733267

**Published:** 2021-10-26

**Authors:** Omar Páez, Pedro Segura-Chama, Angélica Almanza, Francisco Pellicer, Francisco Mercado

**Affiliations:** ^1^Laboratorio de Fisiología Celular, Dirección de Investigaciones en Nuerociencias, Instituto Nacional de Psiquiatría Ramón de la Fuente Muñiz, Ciudad de México, Mexico; ^2^Cátedras CONACyT, Instituto Nacional de Psiquiatría Ramón de la Fuente Muñiz, Ciudad de México, Mexico; ^3^Laboratorio de Neurofisiología Integrativa, Dirección de Investigaciones en Neurociencias, Instituto Nacional de Psiquiatría Ramón de la Fuente Muñiz, Ciudad de México, Mexico

**Keywords:** proton, nociceptor, pain, acid, ASIC, TRP channels, DRG

## Abstract

Pain by chemical irritants is one of the less well-described aspects of nociception. The acidic substance is the paradigm of the chemical noxious compound. An acidic insult on cutaneous, subcutaneous and muscle tissue results in pain sensation. Acid (or H^+^) has at least two main receptor channels in dorsal root ganglia (DRG) nociceptors: the heat receptor transient receptor potential vanilloid 1 (TRPV1) and the acid-sensing ionic channels (ASICs). TRPV1 is a low-sensitivity H^+^ receptor, whereas ASIC channels display a higher H^+^ sensitivity of at least one order of magnitude. In this review, we first describe the functional and structural characteristics of these and other H^+^-receptor candidates and the biophysics of their responses to low pH. Additionally, we compile reports of the expression of these H^+^-receptors (and other possible complementary proteins) within the DRG and compare these data with mRNA expression profiles from single-cell sequencing datasets for ASIC3, ASIC1, transient receptor potential Ankiryn subtype 1 (TRPA1) and TRPV1. We show that few nociceptor subpopulations (discriminated by unbiased classifications) combine acid-sensitive channels. This comparative review is presented in light of the accumulating evidence for labeled-line coding for most noxious sensory stimuli.

## Introduction

Nociception is the nerve coding for noxious stimuli. In the last two decades, many advances have been made to understand how different nociceptive stimuli are transduced, which nociceptors are involved in their transduction, and how afferent neurons relay the information to second-order neurons in the dorsal horn of the spinal cord (Abrahamsen et al., 2008; Moehring et al., 2018; Emery and Wood, 2019; Emery and Ernfors, 2020). Nociceptors respond to a variety of stimuli that can be classified into three categories: (i) mechanical (high-threshold mechanical stimuli), (ii) chemical (pruritogenic compounds, acidic substances, etc.), and (iii) physical (high or low temperature). Most nociceptors respond to a specific kind of nociceptive stimulus, remaining unresponsive to other noxious stimuli (Cavanaugh et al., 2009; Emery et al., 2016). This conclusion challenges a long-lasting dominant idea in which most nociceptors are considered polymodal (Perl, 1996), demonstrating that nociceptors transduce stimuli in a labeled line-code fashion. Using next-generation research techniques, it is now possible to identify most of the afferent neurons transducing distinct noxious stimuli.

Noxious-cold nociceptors are characterized by the expression of TRPM8 channels, corresponding to approximately 10–20% of nociceptors. TRPM8 is an ionic channel that is opened by cold temperatures (<10°C), allowing flow of a cationic current that depolarizes the neuron. There is still controversy about whether TRPM8 is the unique channel necessary for noxious cold transduction since, for example, transient receptor potential Ankiryn subtype 1 (TRPA1) could have a role in cold sensing (Story et al., 2003). TRPM8 knockout (KO) mice have an important behavioral deficit in sensing cold environments; they poorly discriminate a cold room from a warm room, but surprisingly, TRPM8 is not necessary for the detection of extreme cold temperatures (<0°C), which activate another subset of nociceptors lacking TRPM8 expression (Dhaka et al., 2007; Luiz et al., 2019).

**Figure 1:**
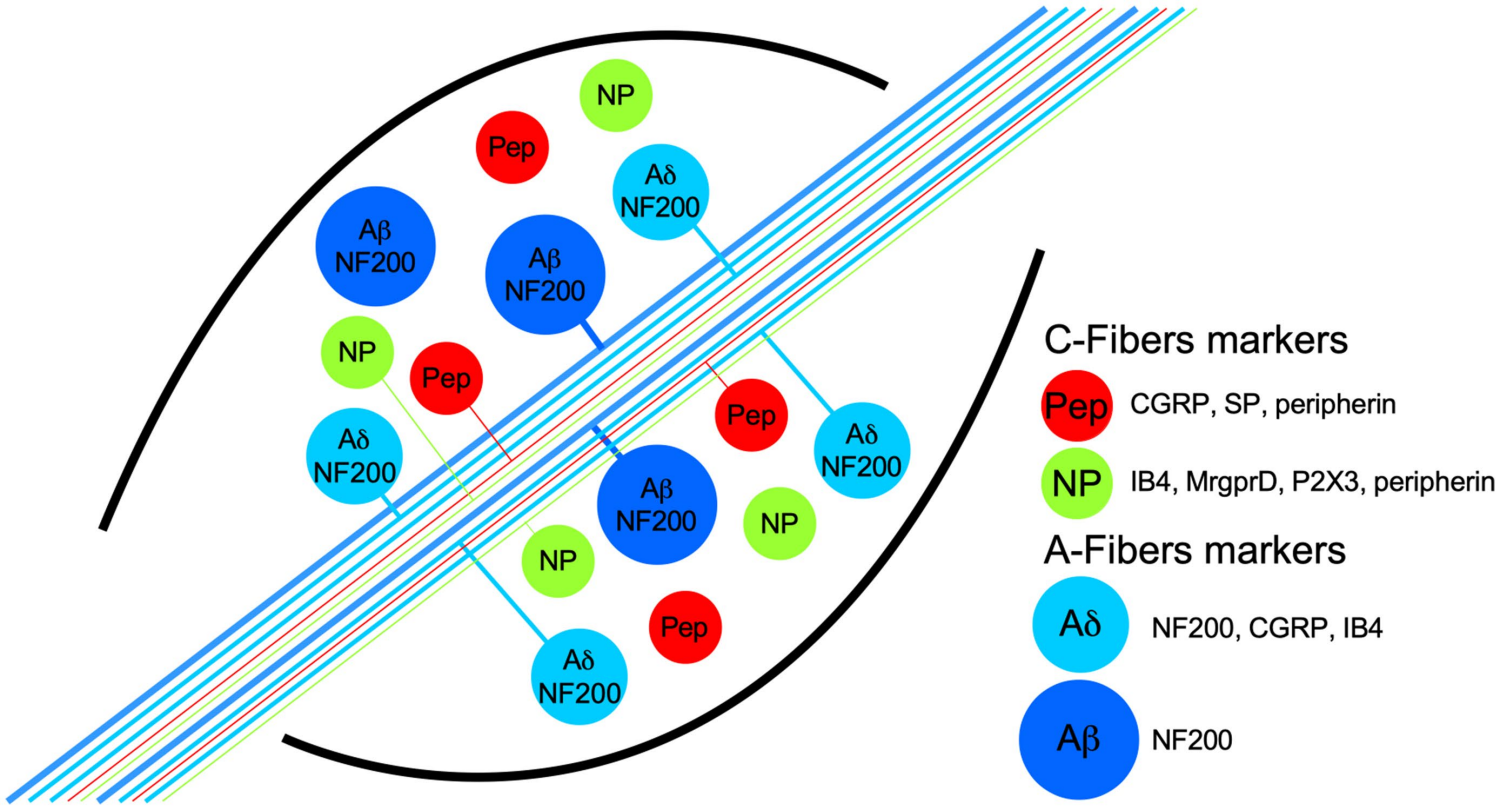
Schematic representation of dorsal root ganglia (DRG) neuron populations classified by their biochemical markers and membrane topology of some proton receptors coupled to an ion channel. The neurons have pseudomonopolar shapes and vary in soma size and axon diameter. Thin, unmyelinated axons belong to small-diameter soma neurons and are called C-fibers (green and red somas), which are classified grossly in two populations by the expression, in some neurons, of the neuropeptides calcitonin-gene related peptide (CGRP) and substance P (SP), which are called peptidergic (red somas; PEP). Nonpeptidergic neurons (green somas; NP) characteristically bind lectin IB4 and express the MrgprD and P2X3 receptors. PEP and NP neurons are stained by the marker peripherin. C-fibers transduce high-threshold stimuli, and almost all are nociceptors. Myelinated axons belong to neurons with larger soma sizes and are called A-fibers. Because the axon conduction velocities are subclassified in α, β, and δ, only the last two are illustrated. Aβ neurons are light-touch neurons, and the axon conduction velocities are faster than Aδ. Meanwhile, a high proportion of Aδ neurons are nociceptors, and both share medium-weight filaments (NF200) as markers, but some Aδ neurons are also stained by CGRP and IB4.

In a similar way, it is known that heat-thermonociceptive neurons express transient receptor potential vanilloid 1 (TRPV1) channels and complementary TRPML3 and TRPA1 (Vandewauw et al., 2018). TRPV1 opens with temperatures above 42°C (Caterina et al., 1997; Tominaga et al., 1998), and in psychophysical experiments, it matches the temperature considered to produce the burning sensation. TRPV1 is coexpressed with the marker calcitonin gene-related peptide (CGRP; Figure 1). Excitotoxic elimination of TRPV1-expressing neurons with intrathecal infusion of capsaicin reduced the CGRP population by half, with other dorsal root ganglia (DRG) nociceptor populations remaining almost unaltered, such as IB4+ neurons (Cavanaugh et al., 2009, 2011). This means that heat-sensing neurons belong to the peptidergic (PEP) nociceptor population. The ablation of TRPV1-expressing neurons produces a profound deficit in the detection of noxious heat, highlighting the importance of that subset of neurons in this sensory modality.

The recently cloned ion channel TACAN, which transduces noxious mechanical stimuli, is expressed by a particular subset of nociceptors that do not express TRPV1, TRPM8, or TRPA1. In fact, TACAN is mainly expressed in nonpeptidergic nociceptors and colocalizes with the marker IB4, the purinergic ion channel P2X3, and the MrgprD receptor (Figure 1; Beaulieu-Laroche et al., 2020). These markers were previously related to the detection of mechanically noxious stimuli (Cavanaugh et al., 2009).

Therefore, cold-coding, heat-coding, and mechano-coding nociceptors are identifiable and experimentally corroborated *in vivo* whole-animal experiments. As many as 85% of nociceptors respond only to a specific kind of the abovementioned stimuli (Emery and Wood, 2019). However, chemonociception is less well-described because chemical sensitivity is infrequently tested (Emery and Wood, 2019).

Physiologically relevant chemical nociception is caused by acidic substances. The cutaneous and subcutaneous application of acid solutions in humans produces clearly recognizable pain, sometimes referred to as “acid burning” (Jones et al., 2004). Infusion of a pH 5.2 solution into the skin (Steen and Reeh, 1993) and hypodermic infusion of a pH 6.5 solution (and lower pH values; Ugawa et al., 2002) elicit pain in human subjects. Muscle pain can also be elicited with a pH 5.2 intramuscular infusion in humans (Law et al., 2008). In animal models, solutions of pH 6.9 and 6.6 are sufficient to elicit nocifensive behavior in rats (Deval et al., 2008). After two intramuscular injections of pH 4 saline, mice develop hyperalgesia without any tissue damage. After the second application of acid, this hyperalgesia becomes chronic (Sluka et al., 2001; Sharma et al., 2009).

In knockout mice for the different acid-sensing receptors, nociceptors continue responding to an acidic solution challenge, albeit with some deficit, and the KO mouse behavioral response resembles that of wild-type mice (Caterina et al., 2000; Price et al., 2000, 2001). The fact that no acid-insensitive phenotype could be obtained opens the possibility that a combination of H^+^ receptors mediates the transduction of acidic stimuli. Moreover, if there is a subset of nociceptors best suited to transduce acidic solutions as noxious stimuli, it has not yet been studied.

In this review, we first summarize the biophysical, pharmacological, and physiological characteristics of ionic channels that act as H^+^-activated receptors, mainly acid-sensing ionic channels (ASICs) and TRP channels. Then, for each H^+^ receptor, we combine the evidence of its expression among neuronal populations in the DRG from rodents. We include reports of expression by both immunohistochemistry and the distribution of acid-induced currents in cultured DRG neurons. Finally, we contrast these data with mRNA expression profiles from the novel unbiased classifications of sensory neurons. We show that the concomitant expression of H^+^-gated receptors (ASIC3, ASIC1, TRPV1, or TRPA1) in sensory neurons is notably subpopulation-specific, which is suggestive of a specialized sensory role for acid in these subpopulations.

## Asic Channels

**Figure 2:**
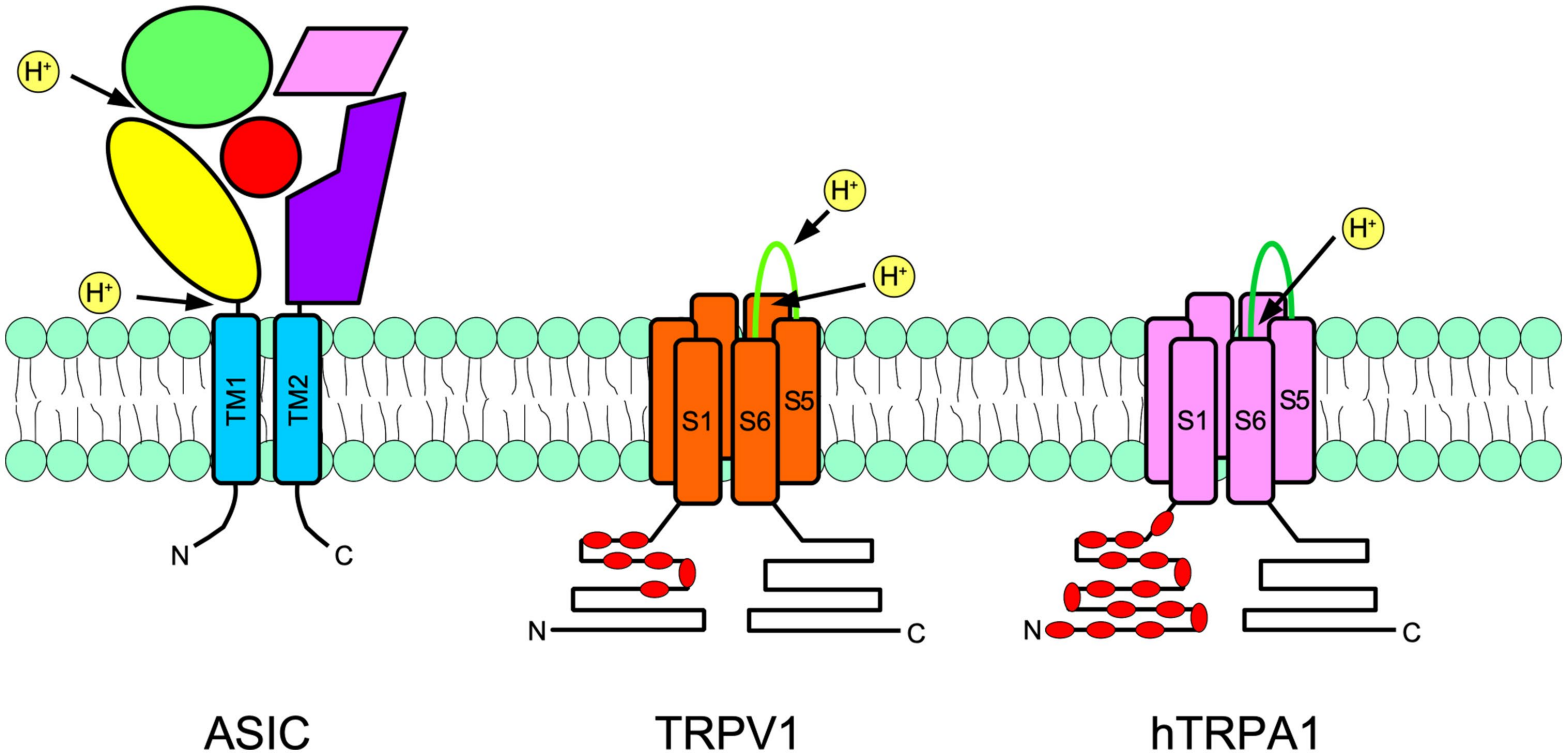
Schematic representation of a subunit of an acid-sensing ion channel (ASIC; left), transient receptor potential vanilloid 1 (TRPV1; center), and transient receptor potential ankyrin 1 (TRPA1; right) are shown. ASIC are trimers, meanwhile TRPs are tetramers. The approximate localization of proton receptors within the protein of each channel is shown with arrows. The ASIC large extracellular portion is arranged in an arm shape, as has been suggested by crystallographic studies. The thumb is in yellow, the palm is in purple, the fingers are in green, beta-balls are in red and knuckles are in pink. In TRPs, red indicates the intracellular ankyrin repeats. N indicates the amino terminus and C indicates the carboxy terminus.

Acid sensing ion channels are homo- or heterotrimeric channels activated by low pH. They are part of the ENaC/degenerin family (Waldmann et al., 1997a,b). There are four ASIC genes that code for six subunits in rodents: ASIC1a, ASIC1b, ASIC2a, ASIC2b, ASIC3, and ASIC4. Each ASIC subunit consists of two transmembrane domains and an arm-shaped conformation. Accordingly, most of the ASIC subunit domains are extracellular, so-called wrist, palm, and thumb domains (Figure 2; Jasti et al., 2007).

Protons bind ASIC subunits *via* an acidic pocket rich in carboxylate groups spanning residues D238–D350, E239–D346, and E220–D408 (Jasti et al., 2007). Outside this pocket, there are also important protonation sites, such as the wrist domain and the first 87 residues after transmembrane domain 1 (Paukert et al., 2008; Li et al., 2009; Schuhmacher et al., 2015). Residues involved in the pH dependence of activation or desensitization are also located in multiple domains spanning the thumb and palm domains (Liechti et al., 2010; Krauson and Carattino, 2016). Activation by protons involves the closure of the thumb domain into the acidic pocket and the opening of the channel gate (Yoder et al., 2018).

When ASIC is activated, an inward Na^+^ current sensitive to amiloride is generated through the channel. The inward Na^+^ current is transient, with fast activation and desensitization kinetics. Desensitization is a nonconducting state that occurs after fractions of a second of prolonged low pH (Waldmann et al., 1997b; Gründer and Pusch, 2015). This desensitized state is controlled by the palm domain (Roy et al., 2013; Vullo et al., 2017). ASIC channels could then be found in closed, open, and desensitized states.

Most ASIC subunits sense pH changes within the physiological range and are activated if the extracellular pH falls below 7, reaching a maximal open probability at pH ~6.0 (Waldmann et al., 1997b; Gründer and Pusch, 2015).

The ASIC subunits (i.e., expressed in Xenopus oocytes) show different pH dependences for activation. Homomeric ASIC3 and ASIC1a channels are the most sensitive to protons. ASIC3 channels show a pH_50_ of 6.4–6.7, and ASIC1a channels show a pH_50_ of 6.4–6.6 (Benson et al., 2001; Sutherland et al., 2001; Babini et al., 2002; Hesselager et al., 2004; Chen et al., 2005; Yagi et al., 2006; Sherwood et al., 2011); moreover, ASIC2 subunits are the least sensitive to acidity (pH_50_ 4.5). ASIC subunits also differ in some other biophysical properties, notably their desensitization time constant, in which ASIC3 and ASIC1a and 1b show significantly faster desensitization kinetics than ASIC2; additionally, ASIC3 shows a significantly larger sustained current at low pH (Hesselager et al., 2004; Salinas et al., 2009; Delaunay et al., 2012). This pronounced sustained current along with its highest sensitivity to pH changes make ASIC3 a strong candidate to transduce tissue acidosis (Dulai et al., 2021). ASICs usually form heteromultimers when coexpressed, so the biophysical properties of heterotrimeric channels reflect their subunit composition (Benson et al., 2001; Hesselager et al., 2004; Gautam and Benson, 2013; Bartoi et al., 2014).

### Distribution of ASIC Currents Among Small Unmyelinated DRG Neurons

The response of DRG neurons to protons has been extensively documented in the literature, and in many cases, the suspected ASIC subunit or combinations of them have been deduced from the current kinetics. In the DRG, nociceptors have been traditionally divided into myelinated medium diameter Aδ and unmyelinated small-diameter C types. Here, we will focus on reports making an explicit distinction between PEP and nonpeptidergic small DRG neurons (Table 1). The common neuronal marker used in these studies is the binding of the IB4 lectin. Most of the neurons not binding IB4 are peptidergic (i.e., they express CRGP), and most of those binding IB4 are nonpeptidergic (Figure 1). These neuronal subpopulations show different currents and action potential characteristics (Stucky and Lewin, 1999; Fang et al., 2006). More importantly, as will be discussed further, these two major groups of nociceptors fulfil different sensory roles within the DRG.

**Table 1:** Distribution of transient ASIC-like currents in small/unmyelinated DRG neurons in culture: peptidergic vs. nonpeptidergic neurons.

Reference	Criteria for cell selection/species	Major division presented here according to	Candidate channel; antagonist used for validation	Peptidergic, IB4−	Non-peptidergic, IB4+
**pH down to 5.2–5.0**					
Liu et al., 2004	Small (15–30μm); rat DRG neurons	IB4-binding	Transient: ASIC channels; blocked by amiloride Sustained: TRPV1 channels; blocked by capsazepine	pH 5.0: 69% mixed currents (transient+sustained)	pH 5.0: 27% mixed currents (transient+sustained)
Drew et al., 2004	Small (<30μm); rat DRG neurons	Wild-type (WT) neurons by IB4-binding	Validated by comparison to ASIC2/3 KO mouse DRG neurons	pH 5.3: 50% transient currents	pH 5.3: 0% transient or mixed currents.
Dirajlal et al., 2003	Mouse DRG neurons that are N52(−) (e.g., unmyelinated) and that have an AP inflection (e.g., nociceptors)	IB4-binding	Transient: ASIC channels; blocked by amiloride Sustained: TRPV1 or ASIC3 channels; also blocked by amiloride at pH 5	pH 5: 33% mixed currents (transient+sustained)	pH 5: 0% transient or mixed currents
Leffler et al., 2006	Small (average 20–24μm); DRG neurons from both mouse and rat	IB4-binding and according to species (WT neurons only)	Transient: ASIC channels; blocked by amiloride	Transient currents:	
				Rat> pH 6.0: 91%; pH 5.0: 78%	Rat> pH 6.0: 40%; pH 5.0: 33%
				Mouse> pH 6.0: 25%; pH 5.0: 19%	Mouse>pH 6.0: 8%; pH 5.0: 3%
**pH down to 6.0**					
Poirot et al., 2006	Small (<30μm); rat DRG neurons	Current type (most to least frequent) and IB4-binding	ASIC1a heteromers according to inhibition by *Psalmopoeus c*. venom and time of course recovery τ	Type 1 current: pH_0.5_ 6.6
~52%	~6%
Likely heteromers containing ASIC1a/3 according to the pH dependence of activation	Type 3 current: pH_0.5_ 6.5	
	~28%	~16%
Likely heteromers containing ASIC2 according to Zn^2+^ positive modulation. Additionally, TRPV1; blocked by capsazepine	Type 2 current: pH_0.5_ 6.0	
	~8%	~10%

When exposed to low pH, some small-diameter rat DRG neurons respond with nondesensitizing, sustained ionic currents, while others respond with desensitizing or mixed currents (Petruska et al., 2000). Consistent with the presence of ASIC subunits, amiloride blocks the transient current and the transient part of mixed currents, whereas sustained currents are insensitive to amiloride. The latter currents are attributed to TRPV1 channels and are observed at lower pH (appearing at pH 6.1); in contrast, the transient currents are attributed to ASIC channels (appearing at pH 6.8; Petruska et al., 2000).

Approximately 90% of all rat DRG small neurons respond to pH 5 with inward currents (Liu et al., 2004). These currents were classified by their desensitization rate into three types: transient, sustained, or mixed. The frequency and waveform of these currents were different between the IB4+ and IB4− subpopulations. The IB4+ population produced most of the sustained (63%) currents, whereas IB4- neurons produced the most mixed currents (69%; Liu et al., 2004). Transient-only currents were not prevalent in any of the two subpopulations (<20% of total observed currents). These transient inward currents begin to activate at pH 7 and are maximal at pH 5. ASICs likely mediate these transient currents since they were sensitive to amiloride. The activation profile of mixed currents results from transient plus sustained currents. Based on these results, it was suggested that small IB4− rather than IB4+ neurons could play an essential role in acid sensing (Liu et al., 2004). Accordingly, ASIC currents (transient and mixed) are exclusive to small IB4- neurons (Drew et al., 2004).

In mouse DRG neurons, specifically unmyelinated nociceptors, it was reported that ASIC currents are also exclusive to the IB4− peptidergic subgroup and largely absent from IB4+ neurons. Transient-only currents were not detected; only (1) mixed and (2) sustained-only currents were detected, and both types were inhibited by amiloride (Dirajlal et al., 2003). DRG neurons from rats and mice generate ASIC currents mostly in IB4- neurons, and this predominance is seen across a wide acidic pH range (pH 6, 5, and 4; Leffler et al., 2006). These reports indicate that within unmyelinated DRG neurons from both rats and mice, ASIC-like currents can be detected almost exclusively within the peptidergic subgroup.

Poirot et al. (2006) provided a more relevant description of ASIC responses in rat DRG small neurons. Only currents that are initially activated within pH values of 6 and higher were investigated. Small sensory neurons produce a slowly inactivating current (Type 1) and two rapidly inactivating currents (Types 2 and 3). Type 1 current is the most prevalent current, the most sensitive to pH (pH_50_ 6.6) and might be mediated by ASIC1a homomers. Type 2 is the less frequent current and is mediated by heteromers containing ASIC2. Type 3 also shows high proton sensitivity (pH_50_ 6.5) and may be mediated by ASIC1a/ASIC3 heteromers (Poirot et al., 2006). Independent of the type, 91% of IB4− but only 36% of IB4+ neurons expressed an ASIC current. The distribution of type 1 and type 3 currents is heterogeneous among small DRG neurons. Type 1 current is expressed at higher levels in IB4− neurons than in IB4+ neurons (6-fold). Type 3 current is also more common in IB4− than IB4+ neurons (Poirot et al., 2006). These results suggest that within small, likely unmyelinated neurons, ASIC1, and ASIC3 might play specific roles in the IB4− peptidergic subpopulation, in which transient proton-gated currents are highly expressed. In addition to the detailed biophysical characterization of ASIC currents in the work by Poirot et al. (2006), this study provided two important demonstrations: (1) activation of ASICs in small DRG neurons can induce action potentials, and the probability of this induction increases with lower extracellular pH, in the pH 6.8–6.0 range; and (2) most H^+^-gated currents in small DRG neurons are mediated by ASICs, with the notable exception of a small fraction of neurons (approximately 15%) expressing the type 2 current, in which there is a high current component mediated by TRPV1 channels (i.e., blocked by capsazepine).

### Expression of ASIC3 and ASIC1 Channels Among Subpopulations of DRG Neurons

In DRG neurons from mice, ASIC3 is the most abundant subunit, whereas ASIC1b is the least abundant subunit (Schuhmacher and Smith, 2016). Regarding its distribution, ASIC subunits, especially ASIC3, do not show heterogeneous expression in immunohistochemistry studies. ASIC3 is more abundant in large-diameter neurons (Alvarez de la Rosa et al., 2002). ASIC3 is expressed mainly in TrkA+ neurons (Molliver et al., 2005), which is the receptor for nerve growth factor (NGF) and a marker of most peptidergic neurons, with medium Aδ and small C fibers. Small sensory neurons that innervate skeletal muscle express more ASIC3 than those that innervate the skin. Moreover, most ASIC3+ muscle afferents (80%) also express CGRP (Molliver et al., 2005). Another study analyzed the expression of ASIC3 in the DRG of eGFP knock-in mice (Lin et al., 2016). One-third of neurons expressed the ASIC3 subunit. ASIC3 localizes to 50% of large-diameter N52+ neurons and 39% of small-diameter peripherin+ neurons. Small peptidergic CGRP+ and IB4+ neurons show lower ASIC3 expression (27 and 23%; Lin et al., 2016). Thus, the expression of the ASIC3 subunit is higher in myelinated Aβ and Aδ fibers.

Analysis of mRNA levels shows that ASIC3 and ASIC1a/b subunits are the most abundant in the complete DRG (Poirot et al., 2006). In a recent work, Papalampropoulou-Tsiridou et al. (2020) analyzed the distribution of ASIC channels in the mouse DRG using RNAscope probes. This tool is a variant of *in situ* hybridization with higher sensitivity. Neurons were classified by the markers NF200 (marker of medium- to large-diameter myelinated neurons), IB4, and CGRP. None of the five ASIC subunits showed a similar distribution among the three different groups of DRG neurons (Papalampropoulou-Tsiridou et al., 2020). IB4+ neurons showed low expression of ASIC3 (~10% of total neurons) and the highest levels of ASIC2a expression (~70% of total neurons), while ASIC1a and 1b were not detected. This is contrasting to the IB4− subpopulation, in which more than 60% express the ASIC3 subunit and approximately 25% express ASIC1a and ASIC1b. Finally, NF200+ myelinated neurons showed the highest levels of ASIC3 subunit expression.

These differences in mRNA levels agree with the conclusions of the electrophysiological studies previously mentioned. First, they corroborate that ASIC3 is mostly expressed in medium to large NF200+ neurons (Alvarez de la Rosa et al., 2002; Lin et al., 2016). This population corresponds to myelinated Aβ and Aδ fibers. In this population, ASIC1 and ASIC1b are expressed at considerably higher levels (>70%) than in small sensory neurons. In fact, in large-diameter DRG neurons, two slowly and two rapidly inactivating H^+^-gated currents have been detected (Poirot et al., 2006). At least two of these currents have a pH dependence of activation (pH_50_ 6.6) similar to that of the more prevalent type 1 current in small neurons (Poirot et al., 2006). Second, the lack of ASIC1 and the lowest expression of ASIC3 subunits in IB4+ neurons explain the scarcity of transient ASIC currents in IB4+ neurons (Dirajlal et al., 2003; Liu et al., 2004; Leffler et al., 2006). Finally, the coexpression of three ASIC subunit mRNAs per single neuron was also analyzed. The three neuron subpopulations (NF200+, IB4+, and CGRP+) show different combinations of ASIC subunits, indicating that heterotrimeric ASIC channels are the dominant form present in most primary sensory neurons. The asymmetric distribution of ASIC subunits in DRG neurons suggests a specialized response to acidification from specific DRG neuronal groups (Papalampropoulou-Tsiridou et al., 2020).

## Trpv1 Channels

Transient receptor potential vanilloid 1 is the most studied member of the TRP family of ion channels. The main agonist of TRPV1 channels is capsaicin, the pungent compound in chili peppers that evokes the burning pain sensation (Caterina et al., 1997). TRPV1 is also a receptor for endovanilloids, lipids, heat, and protons. Each TRPV1 subunit is formed by a TRP domain and six transmembrane domains, and four subunits form a functional channel (Figure 2). The C and N terminals are located at the intracellular side of the channel, where an ankyrin repeat domain is also located (Liao et al., 2013; Rosasco and Gordon, 2017). TRP channels have cationic unspecific pores, in which Ca^2+^ ions are highly permeable, but significant Na^+^ and K^+^ conductance could be detected (Tominaga et al., 1998; Garcia-Sanz, 2004).

Compared to ASIC channels, TRPV1 channels respond differently to low pH. TRPV1 channels show two responses to protons: (1) sensitization and (2) direct activation. Sensitization occurs at extracellular pH>6 and is observed upon activation by capsaicin and heat (Tominaga et al., 1998; Baumann and Martenson, 2000; Jordt et al., 2000; Neelands et al., 2005; Ryu et al., 2007). Direct activation occurs at lower pH (<6) and results in a sustained current at physiological temperature (Caterina et al., 1997; Tominaga et al., 1998). Protons behave as partial agonists of TRPV1 in comparison to capsaicin (20–30% of the current magnitude at saturating doses; Tominaga et al., 1998). TRPV1 H^+^-gated currents show similar kinetics to those induced by capsaicin. Both presented similar desensitization profiles and are sensitive to the antagonist capsazepine (Tominaga et al., 1998).

Different extracellular residues are responsible for TRPV1 activation and potentiation by protons. For example, Glu600 participates in setting the pH sensitivity of the H^+^-potentiation effect on TRPV1, and Glu648 is involved in direct activation by protons (Jordt et al., 2000). In rat TRPV1, two domains were identified in direct activation by extracellular protons. These domains are the pore helix, especially Thr366, and in the linker (TM3–TM4) region, the residue Val538 (Ryu et al., 2007). In the human TRPV1 channel, Glu536 in the TM3 to TM4 linker is required for the proton potentiation effect of capsaicin at high doses (Wang et al., 2010a). All these residues are different from the capsaicin-binding site, which is located intracellularly between the second and third transmembrane helices (Welch et al., 2000; Jordt and Julius, 2002).

### Distribution of Acid-Induced TRPV1 Currents in Cultured DRG Neurons

Transient receptor potential vanilloid 1 colocalizes with ASIC1a and ASIC3 within single small sensory neurons to a considerable extent (Ugawa et al., 2005). Indeed, several reports of ASIC-like currents in cultured DRG neurons have simultaneously detected H^+^-gated TRPV1-like currents (Neelands et al., 2010; Blanchard and Kellenberger, 2011). In response to protons, mouse DRG neurons produce a sustained inward current that inactivates slowly (>20s; Dirajlal et al., 2003). Small C-type neurons show an unequal distribution of these TRPV1-like H^+^-activated currents. At pH 5, 86% of small IB4− neurons generate sustained and, to a lesser extent, sustained and transient currents. Only 33% of IB4+ neurons responded in a similar way. However, not all neurons generating a sustained TRPV1-like H^+^-gated current are capsaicin-responsive (Dirajlal et al., 2003). In another study, only a quarter of small IB4+ neurons showed sustained currents in response to pH 5.3, and half of IB4− neurons showed either sustained or mixed currents (Drew et al., 2004). As expected, in DRG neurons from double-knockout ASIC2/3 mice, the distribution of these sustained currents is not affected (Drew et al., 2004). This heterogeneous distribution of low pH sustained currents was not observed in cultured DRG neurons from rats (Liu et al., 2004).

Rat DRG neurons are more responsive to capsaicin than mouse DRG neurons and have a higher prevalence and magnitude of H^+^-activated currents. When DRG neurons from TRPV1(−/−) mice were analyzed, there was a high reduction in proton responses. In comparison to WT neurons, TRPV1(−/−) neurons show significantly smaller currents at pH 5 and 4 (Leffler et al., 2006). As expected, transient ASIC-like currents remained unaltered in TRPV1(−/−) neurons. Moreover, in a skin nerve preparation *in vitro*, the response of myelinated Aδ fibers to pH 5 and 4 was unaffected in TRPV1(−/−) neurons in comparison to WT neurons. This suggests that the observed TRPV1 responses to low pH are predominantly generated by small unmyelinated neurons (Leffler et al., 2006).

### Expression of TRPV1 Across the DRG

Cavanaugh et al. (2011) used two lines of knock-in mice to study the distribution of TRPV1 in the DRG. During development, a wide variety of DRG neurons, both peptidergic and nonpeptidergic, express TRPV1 (Cavanaugh et al., 2011). In adult mice, however, TRPV1 expression is more restricted to the peptidergic population. Of the total TRPV1-expressing neurons, 64% were peptidergic, and only 11% were nonpeptidergic IB4+ neurons. Of all SP+ neurons in the DRG, 82% are TRPV1+, and of all CGRP+ neurons, 49% are TRPV1+ (Cavanaugh et al., 2011). Substance P (SP) is a marker of unmyelinated peptidergic neurons, while CGRP marks all peptidergic neurons (Aδ and C fibers). Previous studies reported a predominant expression of TRPV1 in the peptidergic subpopulation (Tominaga et al., 1998; Zwick et al., 2002). Since the detection of reporter genes is a more sensitive tool than immunostaining, the study of Cavanaugh et al. (2011) overcame the underestimation of TRPV1 expression in previous studies (Breese et al., 2005; Hjerling-Leffler et al., 2007). Interestingly, 20% of all TRPV1+ neurons are neither IB4+ nor CGRP+ (Cavanaugh et al., 2011).

Patil et al. (2018) classified DRG neurons based on their electrophysiological properties. The authors assessed DRG neurons in culture from several transgenic mouse lines carrying reporter genes for Nav1.8, TRPV1, and CGRP. They compared these against those of transgenic mice expressing well-established neuronal markers. Four of the resulting groups of neurons expressed TRPV1. The four TRPV1+ groups are small-diameter neurons. Three of those subpopulations are indeed peptidergic (CGRP+), and only one is nonpeptidergic (CGRP-; Patil et al., 2018).

Dorsal root ganglia neurons reveal a gradient of expression of TRPV1. Small neuronal bodies and a range of lightly stained neurons of various sizes are densely stained (Goswami et al., 2014). In the rat DRG, colocalization of TRPV1 with the NF200 marker was reported to occur in 11% of the total TRPV1+ neurons (Mitchell et al., 2014). In the mouse, however, TRPV1-induced reporter genes marked approximately 5% of myelinated NF200+ neurons. Thus, within the peptidergic population, there is one small group of myelinated TRPV1+ neurons (Cavanaugh et al., 2011).

## Transient Receptor Potential Ankiryn Subtype 1

Transient receptor potential Ankiryn subtype 1 channel also plays a role in H^+^ detection in intra- and extracellular compartments. TRPA1 is activated for compounds considered irritants such as mustard oil (MO), acrolein, formaldehyde, and cinnamaldehyde (Jordt et al., 2004; Bautista et al., 2005). Extracellular H^+^ elicits ionic current opening in human TRPA1 in channels but not in rodent orthologs (de la Roche et al., 2013). The response to extracellular pH of hTRPA1 has a pH_50_ of 6.5, which is intermediate between the proton sensitivity of most ASICs and TRPV1. Residues V942 and S943 (in the human channel sequence but substituted by an isoleucine and an alanine in the rodent channel) are important for extracellular H^+^-sensing. The ionic current evoked by H^+^ is similar to those evoked by MO or any other agonist of TRPA1 and is a calcium-permeable current with slow kinetics (de la Roche et al., 2013).

The contribution of TRPA1 to the response to acidic substances at the physiological level has also been proposed. In experiments in rodents, TRPA1 contributes significantly to the detection of weak acids and CO_2_ in trigeminal ganglia, and the response is highly specific to neurons expressing TRPA1 (Wang et al., 2010b, 2011). CO_2_ or weak acid application also acidifies the cytosol, so intracellular protons bind TRPA1, activating it, which depolarizes neurons. Similar results have been obtained by blocking Na^+^-H^+^ exchanger 1 (NHE1), which is the main proton pump expressed by DRG neurons and is important for maintaining intracellular pH homeostasis. NHE1 extrudes H^+^ to the extracellular space, increasing the pH in the intracellular compartment. Intracellular acidification induced by zoniporide (a specific blocker of NHE1) directly activates TRPA1 and inhibits its desensitization, increasing calcium entry during intracellular acidosis in TRPA1-expressing neurons (Martínez-Rojas et al., 2021). In painful conditions, such as inflammatory pain, NHE1 expression is downregulated; consequently, intracellular pH acidification produces sensitization of DRG neurons and hyperalgesia, which is in part prevented by TRPA1 blockers.

### Expression of TRPA1 Across the DRG

Transient receptor potential Ankiryn subtype 1 is expressed by small-DRG neurons. Neurons expressing TRPA1 almost always express TRPV1 channels, and approximately half of the neurons that respond to capsaicin also respond to mustard oil or some other TRPA1 agonist (Bautista et al., 2005). Additionally, TRPA1 has a high degree of colocalization with CRGP, SP, and IB4 (Bautista et al., 2005; Kim et al., 2010). The expression of TRPA1 in medium- or large-diameter DRG neurons was almost absent (less than 6% of large neurons). TRPA1 was found to be functionally expressed by a significant proportion of nonpeptidergic neurons (NP; 80% of TRPA1-responsive neurons to MO bind IB4) but not in a great proportion of CRGP+ neurons (only 20% of CRGP+ neurons responded to MO; Barabas et al., 2012). This last is supported by transcriptomic studies in DRGs, where higher TRPA1 expression was detected in nonpeptidergic neurons than in peptidergic nociceptors (Usoskin et al., 2015; Zeisel et al., 2018).

## Biochemical Markers in Drg Subpopulations and Single-Cell Transcriptomic Analysis

Almost every neuronal marker shows some degree of overlap between neuronal subgroups. The lectin IB4, used for dividing nociceptors in most of the reports of acid-induced currents we have reviewed, is not 100 % accurate. Some subpopulations of nonpeptidergic (CGRP-) but IB4− C fibers exist in the mouse DRG (Patil et al., 2018), and approximately 10% of CGRP+ neurons bind IB4 (McCoy et al., 2013). Furthermore, Price et al. (2000) showed that there are a substantial number of peptidergic (CGRP+) neurons that bind IB4 in rats. This overlap between CGRP and IB4 occurs to a lesser extent in mice (Price and Flores, 2007). The relationship between soma diameter and fiber type, despite reflecting normal distributions, is also overlapping (Lawson et al., 2019).

Neuropeptide expression is another example of overlapping between subtypes. Labeling nociceptors as “nonpeptidergic” does not mean they do not express neuropeptides (i.e., Calca and Tac1 genes). New classifications of mouse DRG neurons have emerged based on single-cell mRNA sequencing overcoming these inherent biases (Usoskin et al., 2015; Zeisel et al., 2018). A combination of markers is encouraged for successful identification of a subtype. Combinations of classical/new markers have already been validated *in vivo*, and a handful of other markers have been proposed (Usoskin et al., 2015).

**Figure 3:**
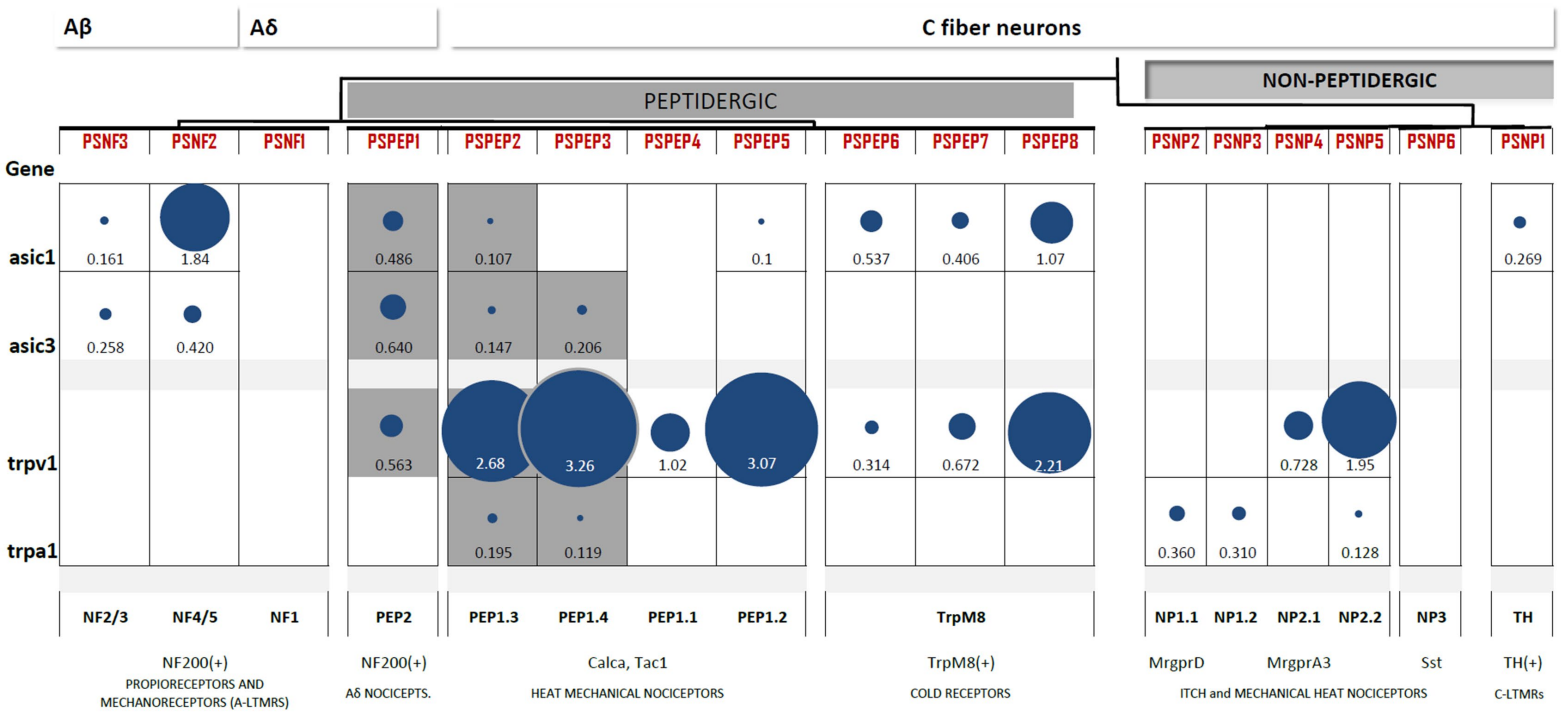
Heterogeneous expression of ASIC1, ASIC3, TRPV1, and TRPA1 channels among subpopulations of DRG neurons. Differential gene expression data generated by Zeisel et al. (2018) available at http://mousebrain.org/genesearch.html. For each gene, numbers are normalized expression levels, and circle size represents relative expression among all clusters. Only those clusters with expression levels above 0.1 for the corresponding gene are shown. Equivalence to clusters reported in Usoskin et al. (2015) is described below, along with some important markers and likely functions per cluster. The subpopulations of nociceptors expressing both TRP and ASIC channels are highlighted in grey.

Extensive evidence has shown that peptidergic C nociceptors are responsible for thermal sensation and pain in response to noxious heat or capsaicin. These heat nociceptors can release neuropeptides and other agents involved in cutaneous inflammation. In contrast, nonpeptidergic nociceptors sense noxious mechanical pain, and some produce itch by chemical irritants (Crawford and Caterina, 2020). This major division of nociceptors in peptidergic and NPs was confirmed in the emerging classifications based on transcriptional profiles. Usoskin et al. (2015) identified 11 groups of neurons: five myelinated A-fiber neurons, of which three are likely LTMRs (NF1, NF2 and NF3) and two are likely proprioceptive (NF4 and NF5). Peptidergic neurons are classified into two groups: unmyelinated C-fibers (PEP1) and lightly myelinated Aδ-type (PEP2; Usoskin et al., 2015). Three groups of nonpeptidergic neurons (NP1, NP2 and NP3) were identified. The clustering of C nociceptors, however, was more comprehensive in the study by Zeisel et al. (2018), which was possible because of the much larger number of neurons sequenced. In the nonpeptidergic population, two subgroups within each of the NP1 and NP2 clusters were identified. In the peptidergic population, PEP1 neurons are classified into four different groups (PSEP2 to PSEP5; Zeisel et al., 2018; Figure 3). That said, there are at least 18 types of neurons in the DRG, and there is a strong correlation between subtype and function (Emery and Ernfors, 2020).

It is worth noting that although this unbiased classification has many advantages, such as the lack of overlapping populations, it is still a picture of the DRG that requires functional validation for most of the resulting clusters; hence, it has opened many opportunities for hypothesis testing regarding the coding of sensory stimuli. Additionally, the conclusions obtained from the search of acid-sensing receptor expression data are complementary to the conclusions obtained with the evidence presented to date.

## Expression of Asic1, Asic3, Trpv1, and Trpa1 in Single-Cell Transcriptomic Analysis

Data on differentially expressed genes in the DRG (Usoskin et al., 2015) and the whole nervous system transcriptome (Zeisel et al., 2018) are publicly available in online datasets.^1^ The contrast between the two clustering systems shows consistency of expression for all genes among groups, despite clustering differences. Here, we collected and compared the expression levels of the most acid-sensitive ASIC subunits, ASIC1 and ASIC3, for TRPV1 and the emerging acid-sensitive TRPA1 channel (Figure 2).

### ASIC1 and ASIC3 Are Not Expressed by Nonpeptidergic Nociceptors

ASIC1 and ASIC3 expression is abundant in two clusters of DRG neurons: NF and PEP. The highest expression of ASIC1 channels is reported in large myelinated Aβ neurons (NF4/5). ASIC1 is also expressed in PEP1 and TRPM8+ cells but is absent from NP neurons. Large myelinated Aβ neurons (NF4/5) also express high levels of ASIC3, which has been linked to proprioceptive mechanotransduction in this population (Lin et al., 2016). However, the highest expression of ASIC3 was found in Aδ nociceptors (PEP2), and similar to ASIC1, ASIC3 was also absent from NP neurons (Figure 3). This largely coincides with the results of mRNA detection showing that neither of the two ASIC1 isoforms, ASIC1a or ASIC1b, can be detected in IB4+ neurons and that ASIC3 expression is lowest in this group (Papalampropoulou-Tsiridou et al., 2020). Contrary to the ASIC1 and ASIC3 subunits, the expression of the least sensitive ASIC2 subunit is almost homogenous across the DRG. Thus, ASIC1 and ASIC3 are not expressed by nonpeptidergic nociceptors, which agrees with electrophysiological, immunohistochemical, and *in situ* hybridization studies.

### TRPV1 Is Characteristic of All Peptidergic Subpopulations, but TRPA1 Is Expressed in Higher Amounts by Nonpeptidergic Neurons

Transient receptor potential vanilloid 1 expression is a feature of all PEP neurons, including C-type nociceptors, Aδ nociceptors, and TRPM8+ neurons. Additionally, TRPV1 was also present in the two groups of NP nociceptors. This pattern of TRPV1 largely coincides with the expected results of electrophysiological (Patil et al., 2018) and reporter gene studies (Cavanaugh et al., 2011). The expression of TRPA1 is very restricted: it is found in specific subpopulations of PEP but mostly in NP neurons. The expression of both TRP channels is negligible in Aβ myelinated neurons. Thus, TRPV1 is characteristic of all peptidergic populations, whereas TRPA1 expression is more restricted (Figure 3).

### Some Peptidergic Subpopulations Combine Acid-Sensitive ASIC and TRP Channels

Only three subpopulations of PEP neurons combine acid-sensitive ASIC and TRP channel expression: PSPEP1 (Aδ nociceptors), PSEP2, and PSEP3 (both mechano-heat noxious neurons; Figure 3). This observation agrees with the findings of the distribution of acid-induced currents, we reviewed in the previous section, in which we concluded that there is a prevalence of acid-induced currents in the peptidergic C-nociceptor population. Thus, based on these two lines of evidence, we hypothesize that peptidergic nociceptors are better suited to transduce H^+^ stimuli than nonpeptidergic nociceptors.

### At the Subpopulation Level, PEP1 Neurons Have Not Been Attributed Specific Sensory Roles

For clarity, in all sections ahead, we will exclude the PSNP6–8 TRPM8+ cluster of cold-related neurons, which are unrelated to acid nociception, and the PSNP1 TH+ cluster of C-LTMRs, as that population does not sense noxious stimuli.

PEP2 (PSEP1) neurons are lightly myelinated Aδ nociceptors. This cluster of Aδ fibers is different from the Aδ-LTMRs innervating longitudinal lanceolate endings (Zimmerman et al., 2014), which are likely represented by the PSNF1 cluster (Figure 3). In addition to the myelin marker NF200, they also express CGRP and the TrkA receptor (Fang, 2005), the latter of which do not stain Aβ myelinated neurons. Aδ nociceptors have some degree of overlap with Aβ fibers with respect to diameter and conduction velocities, and they constitute less than 4% of total DRG neurons (Djouhri and Lawson, 2004; Lawson et al., 2019). Additionally, Aδ nociceptors are considered to be sensors of the “first pain” to noxious heat (which humans perceive as a cold sensation; Treede et al., 1998). Cutaneous Aδ nociceptors sensitize to mechanical stimuli after inflammation, similar to their C-fiber counterparts (Andrew and Greenspan, 1999; Potenzieri et al., 2008). Thus, Aδ nociceptors have a mechano-heat-noxious role as PEP1 C fibers do but with faster conduction velocities.

PEP1 (PSPEP2–5) neurons are unmyelinated nociceptors. As a group, they are defined by the expression of the marker Tac1. This nociceptor group is formed by classical mechano-heat noxious neurons (Emery and Ernfors, 2020). Notably, the subpopulations PSPEP2 and PSPEP3 combine TRPV1 and TRPA1 expression and could represent the main thermal nociceptors (i.e., C-type heat nociceptors) given that the triple deletion of TRPV1+TRPA1+TRMP3 abolishes noxious heat perception (Vandewauw et al., 2018). In addition, it is possible that the so-called “silent nociceptors” could correspond to some subpopulation of the PEP1 cluster. These unresponsive neurons are likely represented by mechanical-insensitive and heat-insensitive type C-neurons (cMiHis) and are present in both PEP and NP nociceptors (Lawson et al., 2019). Silent nociceptors are a group of peptidergic (CGRP+, IB4−, and TrkA+) C neurons expressing CHRNA3 and are sensitized to mechanical stimuli upon inflammation (Prato et al., 2017). Interestingly, the inflammation mediator PGE2 sensitizes C-type mechanically insensitive nociceptors (CMi). Whether thermal and/or silent nociceptors are indeed represented by discrete subpopulations of the PEP1 cluster remains to be demonstrated.

Another possibility is that PEP1 subpopulations could represent different transcriptional states of the same neuronal group, as was considered by Usoskin et al. (2015), to reconcile the evidence for polymodality with the evidence for neuronal diversity. A recent study described the stimulus responsiveness (i.e., mechanical, heat, mechanical-heat, etc.) of DRG neurons innervating cutaneous tissue, in which neurons were clustered based on single-cell transcriptional profiling of 28 selected genes (Adelman et al., 2019). Cutaneous PEP neurons include two main types of nociceptors: C-type heat nociceptors (CHs) and polymodal C-type mechanical and heat nociceptors (CMHs), whereas NP neurons mainly include C-type mechanical nociceptors (CMs) and polymodal CMHs (Adelman et al., 2019).

Both groups of cutaneous nociceptors, PEP and NP, include CMHs, which are the most common type of C-type polymodal nociceptors (cPMNs) in the DRG (Dubin and Patapoutian, 2010; Lawson et al., 2019). Since the majority of CMHs and cPMNs in general are nonpeptidergic IB4+ nociceptors (Rau et al., 2009; Adelman et al., 2019), the role of PEP1 neurons as polymodal nociceptors seems less likely. Previous reports in cutaneous peptidergic neurons (SP+) of mice showed that CMHs also responded to the application of acid (pH 5) *via* ASIC3 (Price et al., 2001).

There are important differences between peptidergic nociceptors innervating muscle and cutaneous tissue. Unlike cutaneous DRG neurons, neurons innervating muscle appear to be clustered in different subpopulations; in particular, the PEP cluster of nociceptors displays more transcriptional variation, suggesting different subtypes in comparison to skin-innervating peptidergic neurons (Adelman et al., 2019). Importantly, more than 50% of Aδ or C muscle afferents (groups III and IV) are chemosensitive, with a very low percentage being polymodal and only 18% of the muscle afferents being silent nociceptors (Jankowski et al., 2013). Chemosensitive neurons innervating muscle are classified into two groups: (1) metaboreceptors, which sense a slight decrease in acidity (pH 7.0), low levels of lactic acid and ATP (Light et al., 2008), and (2) metabonociceptors, which respond to higher levels of lactic acid and ATP and higher acidity (pH 6.6). Both Aδ and C muscle-innervating metabonociceptors were found to be TRPV1+ and ASIC3+, whereas nonnociceptive metaboreceptors were TRPV1− and ASIC3− (Jankowski et al., 2013). This suggests that acid-sensitive Aδ and C muscle nociceptors are likely to be PEP neurons, the only cluster containing subpopulations expressing TRPV1 and ASIC3 together.

Thus, the assignment of distinct functional roles among PEP subpopulations is further complicated by the different innervated target tissues. It is more frequent for peptidergic (CGRP+) fibers to terminate in deeper tissues than nonpeptidergic (IB4+ or MrgprD+) fibers (Zylka et al., 2005). Accordingly, the majority of visceral afferents are peptidergic (CGRP+; Robinson and Gebhart, 2008), and the majority of silent nociceptors also innervate deep tissues (Prato et al., 2017). In fact, it has been argued that the PSPEP2 subpopulation might innervate cutaneous tissue, and the PSPEP3 subpopulation innervates deeper tissues (Emery and Ernfors, 2020).

ASIC3 and TRPV1 were coexpressed in only three peptidergic subpopulations of DRG neurons. PSEP1 neurons have a defined role as myelinated nociceptors and are susceptible to sensitization by inflammation. PSEP2 and PSPEP3 neurons likely represent heat nociceptors but, since they are part of the PEP1 cluster, may include mechanical-heat polymodal fibers, especially in the case of cutaneous afferents. In muscle afferents, it is possible that the PSEP2 subpopulation senses noxious acidosis.

## Conclusion

In this work, we reviewed the characteristics of proton-gated ion channels, which once exposed to low pH increase the neuronal excitability in DRG nociceptors. ASICs and TRP (particularly V1 and A1 subtypes) are strong candidates for proton sensing in the somatosensory system. Experimental evidence about the nocifensive response against acid insult showed that most of these proteins participate in acid sensing, but as previously mentioned, abolishing only one of them does not produce an acid insult-insensitive phenotype.

The pattern and high titers of expression of proton-gated channels are restricted to a subset of PEP small-diameter neurons. This is intriguing since it suggests that acid sensing in nociceptors might be coded in a labeled-line fashion. Whichever the case, the selective ablation of some peptidergic subpopulations could give us a phenotype unresponsive to acidic insults.

Since chemical nociception is the less well-described nociceptive modality, it is important to complete the picture of how nociceptors are activated. It is not documented that the population responsible for activation by an acidic insult is worth finding. It will be interesting to determine whether acid detection by nociceptors obeys labeled-line coding, as occurs for noxious-mechanical or noxious-heat stimuli. Once identified, the physiology of acid-activated nociceptors could be better studied during nociceptive pain and better characterized during the genesis or maintenance of pathologically painful conditions.

## Author Contributions

OP collected the bibliographic information, elaborated the figure, and conceived and wrote the manuscript. PS-C reviewed the bibliographic information and the manuscript. AA conceived and reviewed the final version of the manuscript. FP reviewed the manuscript. FM supervised the work, collected bibliographic information, and conceived, wrote, and reviewed the final version of the manuscript. All authors contributed to the article and approved the submitted version.

## Funding

This work was supported by an INPRFM grant (NC12165994.0) to FM.

## Conflict of Interest

The authors declare that the research was conducted in the absence of any commercial or financial relationships that could be construed as a potential conflict of interest.

## Publisher’s Note

All claims expressed in this article are solely those of the authors and do not necessarily represent those of their affiliated organizations, or those of the publisher, the editors and the reviewers. Any product that may be evaluated in this article, or claim that may be made by its manufacturer, is not guaranteed or endorsed by the publisher.

## Footnotes

^1^ http://linnarssonlab.org/data/
